# The Nuts and Bolts of Implementing a Modified ERAS Protocol for Minimally Invasive Colorectal Surgery: Group Practice vs. Solo Practice

**DOI:** 10.3390/jcm11236992

**Published:** 2022-11-26

**Authors:** Zhen-Hao Yu, Yih-Jong Chern, Yu-Jen Hsu, Bor-Kang Jong, Wen-Sy Tsai, Pao-Shiu Hsieh, Ching-Chung Cheng, Jeng-Fu You

**Affiliations:** Division of Colon and Rectal Surgery, Chang Gung Memorial Hospital, College of Medicine, Chang Gung University, Linkou, Taoyuan 33305, Taiwan

**Keywords:** colorectal cancer, enhanced recovery after surgery, group practice, minimally invasive surgery

## Abstract

**AIM:** The ERAS protocol consists of multiple items that aim to improve the outcomes of patients receiving surgery. Adhering to the protocol is difficult. We wondered whether surgeons practicing the ERAS protocol in a group would improve patient outcomes. **Methods:** All patients who underwent colorectal resection for benign disease or malignancy from November 2017 to December 2018 were collected and reviewed retrospectively. According to the physician’s ward round strategy, the patients were categorized into two groups, either by solo practice or group practice. **Results:** This study enrolled 724 patients and divided them into two groups according to the practice method: group practice (*n* = 256) and solo practice (*n* = 468). The group practice cohort had less postoperative morbidity (14.0% vs. 21.4%, *p* = 0.048) and shorter postoperative hospital stays (mean: 6.6 ± 3.2 vs. 8.6 ± 5.5, *p* < 0.05) than the solo practice cohort. Group practice (*p* < 0.001), natural orifice specimen extraction (NOSE) procedure (*p* < 0.001), and blood loss >50 mL (*p* = 0.039) significantly affected discharge within 5 days postoperatively in multivariate analyses. **Conclusions:** Group practice based on a modified ERAS protocol shortens postoperative hospital stays with fewer morbidities compared with solo practice in which patients receive elective minimally invasive colorectal surgery.

## 1. What Does This Paper Add to the Literature?

ERAS checklists for minimally invasive surgery have recently been shown to have a distinct impact on recovery in patients with CRC. However, the influence of medical practice on ERAS compliance is unknown. Here, we show that group practice based on a modified ERAS protocol shortens hospital stays compared to solo practice.

## 2. Introduction

Enhanced Recovery After Surgery (ERAS^®^) is a protocol to improve the results of patients who receive all aspect of colorectal surgery. It is based on published evidence and has been found to shorten the length of hospital stay and decrease postoperative morbidities, costs, and readmissions following colorectal surgeries [1].

The ERAS protocol for colorectal surgery consists of multiple concepts and standardized peri-operative strategies and practices, including preadmission education and peri-operative guidance during admission [2]. The success of executing the protocol requires the collaboration of each team member, including surgeons, nurses, nutritionists, and anaesthesiologists.

Although multiple peri-operative ERAS components currently exist, the thorough implementation of the ERAS protocol is essential to improve patient recovery after surgery [3,4]. Traditionally, colorectal surgeons prefer to manage patients by administering an oral diet step by step, from sips of water to clear liquids, full liquids, and to a soft diet after bowel function is restored. Even after the ERAS protocol was introduced, surgeons still delayed patients’ oral intake, despite evidence of the advantage of early feeding [5]. Thus, it is crucial to increase adherence to the ERAS protocol [6,7].

After the first group practice set up by the Mayo brothers in the mid-1880s, doctors around the world formed various groups to offer services to their patients [8,9]. Although the reasons why these groups were formed may differ, group practices shared some unique characteristics. Group practices improve patient satisfaction and experiences by lowering wait times and increasing access to care. Group practices also improve the quality of care by increasing adherence to guidelines through more convenient knowledge sharing and access to information between group members [10]. These standardized guidelines ensured continuity of care for the patients. Group practice also offers benefits for physicians, such as increasing quality of life and satisfaction, higher competency and financial gain, and decreasing burnout. These advantages come from better work–life balance, shared call responsibilities, improved knowledge transfer, collaboration and decreased professional isolation [11]. Positive interpersonal relationships and a common vision also aid the well-being of group practice members.

Currently, it is unclear whether the style of medical practice affects the implementation of ERAS for patients with colorectal cancer (CRC). There are two common types of medical practice: solo practice and group practice. Solo practice is a standard and effective method led by an attending surgeon, including outpatient diagnosis and treatment, surgery, and related care after surgery [11]. A group practice is guided by multiple attending surgeons who may provide all clinical care among members of the team.

After the Mayo brothers started their first group in the mid-1880s, physicians worldwide formed various groups that desired to provide the best services for their patients. Although these groups are constructed differently, group practices share similar characteristics. First, group practice improves patient satisfaction and experience by reducing wait times and increasing access to care. Second, group practice also increases the quality of care by increasing adherence to guidelines through easier knowledge sharing and access to information among group members [12]. In addition, physicians can benefit from improved quality of life and satisfaction, increased competence, shared on-call responsibility, improved knowledge transfer, collaboration, and reduced professional segregation and burnout [13].

This study aimed to clarify whether the type of medical practice affects the adherence rate of the ERAS protocol for minimally invasive colorectal surgery. We evaluated the short-term outcomes of patients who underwent colorectal surgery following a modified ERAS protocol by group-practice surgeons compared with single-practice surgeons at a single tertiary care centre.

## 3. Methods

### 3.1. Study Design and Patient Selection

Detailed information on patients who underwent elective colorectal resection for benign disease or malignancy in a single medical institute, Chang Gung Memorial Hospital, from November 2017 to December 2018 was collected prospectively and reviewed retrospectively. All the data came from patients’ medical records, and we obtained informed consent from all patients for use of their data in this study. The institutional review board approved this study (IRB No.202201164B0).

The inclusion criteria were (1) a segment of bowel resection, including colon and rectum resection, (2) minimally invasive surgery, and (3) an American Society of Anaesthesiologists (ASA) physical status score less than or equal to 3. Patients who received laparotomy and conversion of laparoscopic to open surgery were excluded.

### 3.2. Solo Practice and Group Practice

Solo practice is a traditional ward round led by a single attending surgeon with fellows, residents, or nurse practitioners. The single surgeon supervises and is responsible for all aspects of a patient’s care, including clinics, admission, surgical intervention, and postoperative care.

Group practice in this study refers to multiple physicians with single specialties in colorectal disease, including four attending surgeons.

The difference between solo and group practice is primarily regarding the ward round and decision making. The single attending surgeon would provide one ward round per day, including weekdays and weekends, to the patients in the solo practice. In group practice, four attending surgeons would take turns providing one ward round per day when his or her clinical workload permitted. Each group practice surgeon would see his or her patients and those of the other three physician’s patients. More information about the solo and group practice is provided in Table S1.

### 3.3. The Modified ERAS Protocol

We did not entirely apply all the ERAS protocol components in our institute because no standardized multidisciplinary consensus has yet been achieved about implementing the whole ERAS protocol [14]. The comparison of the perioperative care protocol between the standard and modified ERAS guidelines is described in Table 1. The main differences are in the administration of carbohydrate loading and drinking clear liquids 2 h before the induction of anaesthesia, multimodal analgesia, early postoperative oral intake, and the use of intra-abdominal and pelvic drains. The main difference between solo and group practice is that the group practice physician relied on the modified ERAS protocol checklist.

### 3.4. Outcomes and Covariables

Measurement outcomes included short-term postoperative complications, recovery, pain score, and readmission. Postoperative complications were defined as morbidity or mortality occurring within 30 days and were graded according to the Clavien–Dindo classification.

Postoperative recovery evaluation was based on blood test reports, pain intensity, and the length of hospital stay. We also collected postoperative 30-day hospital readmission data. Pain intensity was estimated using a numeric rating scale (NRS) from 0 to 10, with 10 indicating the worst unbearable pain. The mean postoperative pain scores of patients were used for further evaluation.

### 3.5. Statistical Analysis

All data analyses were performed using IBM SPSS Statistics, Version 24.0 (IBM Corp., Armonk, NY, USA). Clinicopathological characteristics with categorical variables are shown as frequencies and proportions and were compared using the chi-square test. Continuous variables are presented as the means and standard deviations and were analysed using Student’s *t* test. Univariate and multivariate analyses were performed using binary logistic regression. A two-tailed *p* value < 0.05 was considered statistically significant.

## 4. Results

**Figure 1:**
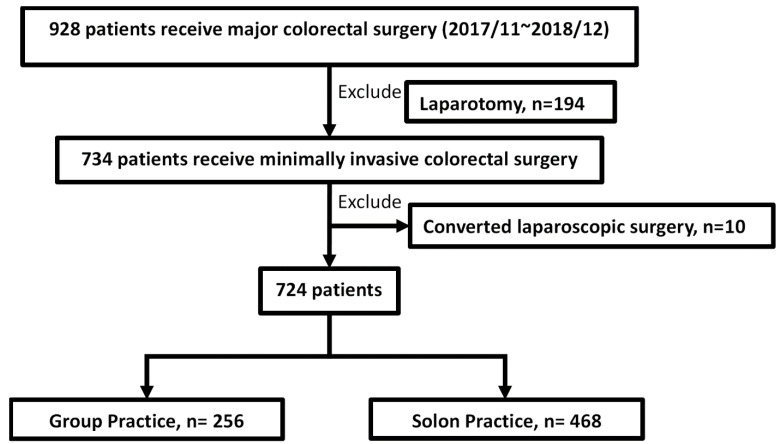
Flow chart of the patient enrolment process for this study cohort.

From November 2017 to December 2018, a total of 928 patients received major colorectal surgery at Chang Gung Memorial Hospital. There were 194 patients who underwent laparotomy and 10 patients who tried laparoscopic surgery first but then converted to laparotomy. This study enrolled 724 patients and divided them into two groups according to practice method: group practice (*n* = 256) and solo practice (*n* = 468) (Figure 1). The demographic data of these patients are presented in Table 1. The two groups did not differ significantly in terms of age, sex, or BMI. The group practice patients had a higher rate of ASA score 3 than the solo practice patients (group practice vs. solo practice, 73.8% vs. 64.7%, *p* = 0.037). The preoperative blood tests for white blood cell (WBC) count, CRP and CEA were similar in both groups, but haemoglobin and albumin levels were slightly higher in the solo practice patients. There was no difference in previous abdominal surgery and neoadjuvant therapy in either group.

Table 2 shows the operative and postoperative data. There were no differences in operative procedure, combined surgery, blood loss, surgical time, diagnosis, or cancer stage between the two groups. The group practice patients had a higher natural orifice specimen extraction (NOSE) rate (29.7% vs. 22.9%, *p* = 0.043), a higher robotic-assisted method (9.0% vs. 0.4%, *p* < 0.05), less postoperative morbidity (14.0% vs. 21.4%, *p* = 0.048), and a shorter postoperative hospital stay (mean: 6.6 ± 3.2 vs. 8.6 ± 5.5, *p* < 0.05) than the solo practice patients. Regarding the postoperative blood test, the group practice patients had a higher WBC count (9.65 ± 3.24 × 10^3^/dL vs. 8.67 ± 4.03 × 10^3^/dL, *p* < 0.05) and haemoglobin level (11.37 ± 2.09 vs. 10.87 ± 3.72, *p* = 0.021). The pain score was also lower among the group practice patients than the solo practice patients postoperatively (2.25 ± 0.77 vs. 2.36 ± 0.68, *p* = 0.048).

The results of univariate and multivariate analyses of discharge within 5 days postoperatively are shown in Table 3. Group practice (OR = 2.810, 95% CI: 2.022–3.905; *p* < 0.001), NOSE procedure (OR = 3.790, 95% CI: 2.662–5.396; *p* < 0.001), operative method, especially low anterior resection (OR = 0.610, 95% CI: 0.374–0.994; *p* = 0.047), neoadjuvant therapy (OR = 0.445, 95% CI: 0.228–0.868; *p* = 0.018), tumour > 4 cm (OR = 0.675, 95% CI: 0.481–0.949; *p* = 0.024), blood loss > 50 mL (OR = 0.388, 95% CI: 0.214–0.703; *p* = 0.002), preoperative CEA > 5 (OR = 0.503, 95% CI: 0.332–0.762; *p* = 0.001), and preoperative CRP > 5 (OR = 0.593, 95% CI: 0.398–0.883; *p* = 0.010) were associated with discharge within 5 days postoperatively in univariate analysis. After multivariate adjustment, group practice (OR = 2.836, 95% CI: 1.985–4.051; *p* < 0.001), NOSE procedure (OR = 3.488, 95% CI: 2.333–5.096; *p* < 0.001), and blood loss > 50 mL (OR = 0.504, 95% CI: 0.263–0.965; *p* = 0.039) were still significant factors affecting discharge within 5 days postoperatively.

**Figure 2:**
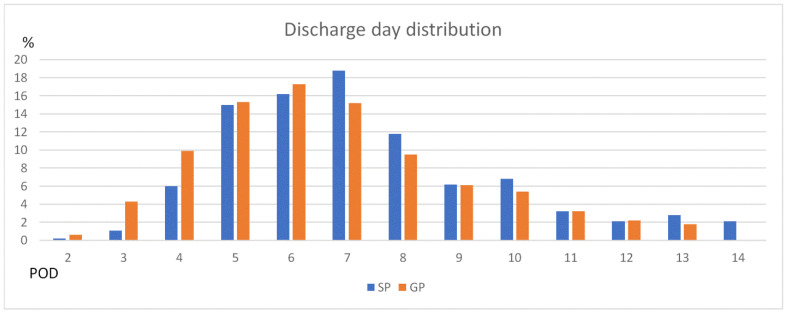
Histogram showing the distribution of postoperative hospital stay for the study population according to solo practice (SP) and group practice (GP)**.** POD = postoperative day.

The discharge day distributions of the group practice and solo practice patients are shown in Figure 2. The mean days of hospital stay were 6.6 ± 3.2 days and 8.6 ± 5.5 days for the group practice and solo practice groups, respectively. The group practice patients had a higher proportion of discharge days at POD 3 and POD 4 than the solo practice patients (4.3% vs. 1.1% and 9.9% vs. 6.0%, respectively). The solo practice group had a higher proportion of discharge days at POD 7 and POD 8 than the group practice patients (18.8% vs. 15.2% and 11.8% vs. 9.5%, respectively).

## 5. Discussion

This study assessed the short-term outcomes of group practice patients based on a modified ERAS protocol compared with solo practice patients. Our data showed that group practice patients had shorter hospital stays and fewer surgical complications than solo practice patients.

Group practice is one of several kinds of medical practices that include solo practice, employed physician practice, and direct primary care [15]. This study is about two practices in our institute, group practice and solo practice. Group practices have been around for more than 100 years, and one of the earliest was established by the Mayo brothers in the 1880s [8,16]. Many studies published in recent years have assessed the pros and cons of this type of medical practice [17,18,19], such as the comprehensive inventory of the pros and cons of group practice in 2021 by Zwiep et al. [11] Among such studies, several benefits have been reported, such as improved patient satisfaction and experience, improved quality of care, and reduced healthcare costs; further, physicians experienced improved quality of life and satisfaction, improved competency, increased income, and improved health information uptake [10,19,20,21,22,23].

We started our group practice, with multiple physicians in a single specialty in 2017. The group initially consisted of four attending surgeons, and later, several younger attending physicians joined. Each day, one of four staff members takes turns conducting ward rounds, giving orders in the morning and setting daily goals for patients. The original attending physicians would later visit the patient whenever possible. In this way, staff avoided splitting their time among ward rounds, clinics, operating rooms, and examinations and delaying patient care. In addition, the team built an excellent electronic medical record platform for shift handovers that rotating residents updated. Based on the medical records and documentation, the staff can exchange various opinions with team members through this platform. Overall, team members were satisfied with this system of group practice. Based on our real-world experiences, this group practice is safe and effective in performing the modified ERAS protocol for minimally invasive colorectal surgery. It is up to team members, not necessarily the individual patient’s surgeon, to make objective decisions based on daily ERAS goals that prevent individual subjective limitations. The results may align with the tenets of objective and protocol-based patient care. However, we did not assess patient satisfaction during this period.

Enhanced recovery after surgery (ERAS) is an evidence-based multispecialty and multidisciplinary approach to surgical patient care that must involve multiple professionals [1,2]. Danish surgeon and professor Henrik Kelhet first proposed this concept for colonic resection in 1953 [24,25,26,27]. These concepts were developed as a well-known ERAS protocol by Ken Fearon and Olle Ljungqvist in 2001 [28,29].

The ERAS society was then formed to develop perioperative care and improve recovery through research, education, auditing, and implementation of this evidence-based program [2]. Currently, the program is accepted worldwide in the colorectal surgical field and other specialties, such as gynaecology, chest surgery, and urology.

The latest guidelines for the ERAS program released in 2018 have a total of 24 items, which are categorized into preadmission, preoperative, perioperative, and postoperative periods [2].

However, the real-world application of ERAS is bound to have modifications. Our institution also has certain limitations, so we applied and modified several program elements at various phases, as shown in Table 1. First, the anaesthesiologist advised all of our patients to fast for at least eight hours before the operation due to concerns about vomiting and aspiration, which is different from the standard ERAS guideline. According to the standard ERAS guidelines, a non-alcoholic clear liquid diet is permitted even 2 h before surgery and a bland meal 6 h before surgery.

Brady et al. [30] reviewed the issue of preoperative fasting systemically and briefed that preoperative water intake reduces residual gastric volume without pulmonary morbidities. In addition, Nygren J reported that preoperative administration of oral carbohydrates decreased overnight fasting and surgery-related catabolism [31]. Furthermore, the Preoperative Oral Carbohydrate (PROCY) trial demonstrated that preoperative carbohydrate administration might improve the perioperative condition, reducing postoperative insulin resistance and protein breakdown [32].

In this study, the mean postoperative hospital stay was 6.6 days in the group practice arm and 8.6 days in the solo practice arm. Compared to other investigations, this study’s hospital stay duration seems to be delayed by approximately 1 to 2 days [33,34]. We suppose that preoperative fasting might be one of the reasons for delayed recovery of bowel function and thus delayed discharge from the hospital.

Second, our pain control strategies are also different from the guidelines. According to the guidelines, multimodal analgesia combined with epidural analgesia and avoiding opioids are suggested [35,36]. In our practice, epidural pain control is not a choice. Instead, we inject a local anaesthetic (bupivacaine 100 mg/20 mL mixed with epinephrine 0.1 mg) at the end of the operation near surgical wounds, including trocar wounds and sites where specimens were extracted. Options for postoperative pain relief include oral analgesics, patient-controlled analgesia, and intermittent intramuscular analgesics.

Third, we usually use surgical drains and Foley catheters. The Jackson-Pratt drain is generally placed over the pelvis for left-sided colectomy, and the Morrison pouch is used for right-sided colectomy. The primary purpose is to evacuate postoperative collections, bleeding, and residual accumulation of gas by laparoscopic pneumoperitoneum. The drain is removed approximately 2 to 3 days after surgery. Once the patient can get out of bed and move well, the Foley catheter can be removed. According to the essence of the standard ERAS protocol, routine drainage is not recommended [37].

A fundamental basis for the success of ERAS implementation is the compliance rate of implementers. The tendency of traditional physicians to give medical orders during ward rounds seems to be related to habit and experience. Some ERAS programs were challenging for traditionally trained surgeons to follow, which may account for the slower recovery of patients in the solo group in this study. The attending surgeons commonly work with patients operated on by other members of the group practice. They give medical orders more often based on the ERAS checklist, which can increase adherence to the ERAS guidelines.

Natural orifice specimen extraction (NOSE) is the opening of a hollow viscus that is already communicating with the outside world, such as the vagina or distal gastrointestinal tract, to remove the surgical specimen [38,39]. In our institute, we usually use the distal gastrointestinal tract (rectum) as the conduit for specimen removal despite the operation site. Tumour size and clinical T staging are 2 major factors for performing the NOSE procedure. We perform this procedure if the short axis of the tumour is less than 4 cm. The contraindication of this procedure is clinical T4 disease because of potential intraluminal tumour cell seeding, which might result in local recurrence. Another issue is increased infection because of the intracorporeal opening of the intestinal tract. Adequate irrigation during surgery might prevent this condition. The benefits of the NOSE procedure include a better postoperative pain score, faster restoration of bowel function, shorter length of hospital stay, and improved cosmesis [40].

In the future, we may set clear discharge criteria and inform patients before admission [41]. After the patient fulfils these criteria, discharge can be arranged confidentially and safely. We will continue discussions with anaesthesiologists about preoperative fasting strategies to shorten the patient’s NPO time, leading to faster restoration of bowel function and normal oral intake.

This study has several limitations. First, the retrospective design was the principal weakness of the current research, and the decision to adopt the modified ERAS protocol was not random. Second, we could not evaluate the adherence rate of each component of the modified ERAS protocol. Third, there is a high variation in physician decisions, especially in the solo group, because our institute employs more than ten attending surgeons. Each physician has his or her personal preferences in decision-making, which causes modified ERAS components to be inconsistent. Fourth, there are still some differences between the modified ERAS protocol and the actual ERAS protocol, especially concerning carbohydrate loading before surgery. Fifth, the surgical techniques and strategies adopted by surgeons, such as robotic surgery and the NOSE procedure, might have some influence on the outcomes and the length of the hospital stay for patients.

## 6. Conclusions

In conclusion, a group practice based on a modified ERAS protocol reduces hospitalization length of stay and improves outcomes without increasing morbidity in patients undergoing minimally invasive colorectal surgery. According to our experiences, group practice can enhance ERAS performance adherence and prevent old habits and empirical tendencies of traditional surgical doctrines that occur in solo practice.

## Figures and Tables

**Table 1:** Comparison of perioperative care protocol between standard and modified ERAS guidelines.

Primary Component	Standard	Modified
*Preadmission*		
Preadmission counselling	Information about preoperative education, surgical indication, and discussion of milestones and discharge criteria	The same
Preadmission optimization	Prehabilitation	The same
*Preoperative interventions*		
Preoperative nutrition	Drink clear fluids continuously <2 h before the induction of anaesthesia Carbohydrate loading should be encouraged before surgery in nondiabetic patients	NPO at least 8 h before surgery Nutritionist referral and parenteral nutrition supplement if needed
Management of anaemia	Not mentioned	Blood transfusion to keep hemoglobin (Hb) > 10 gm/dL
Mechanical bowel preparation	Recommended	Commonly used except for nearly obstructive tumours and elderly patients
Oral antibiotic preparation	Recommended	Not routine
*Perioperative interventions*		
Reduce surgical site infection	Preparation of surgical field with chlorhexidine Prophylactic antibiotics before incision	2% chlorhexidine as antiseptic Cefazolin 1 gm + metronidazole 500 mg within 30 min of incision
Prevention of nausea and vomiting	Combination of ondansetron with dexamethasone before anaesthesia	Ondansetron before anaesthesia
Intraoperative fluid management	Avoid volume overload Balanced chloride-restricted crystalloid solutions should be used as maintenance Goal-directed fluid therapy	The same
Pain control	A multimodal, opioid-sparing, pain management plan before the induction of anaesthesia Transversus abdominis plane block with a local anaesthetic Thoracic epidural analgesia is recommended for open colorectal surgery, but not for routine use in laparoscopic colorectal surgery	Local anaesthetic at the end of surgery Multimodal pain control
Surgical approach	A minimally invasive surgical approach should be used	All minimally invasive surgery included in this study
Use of intra-abdominal drains and nasogastric (NG) tubes	Should not be routinely used	Remove NG tubes at the end of surgery Jackson-Pratt drain is optional
*Postoperative interventions*		
Patient mobilization	Early and progressive patient mobilization	As soon as possible but not compulsive
Ileus prevention	A regular diet immediately after elective colorectal surgery	Progress gradually from clear liquid diet to full liquid diet and then soft diet according to patient’s condition and physician’s decision
Postoperative fluid management	Intravenous fluids should be discontinued in the early postoperative period	Discontinued if patient intake is smooth
Urinary catheters	Urinary catheters should be removed within 24 h of elective colonic or upper rectal resection when not involving a vesicular fistula. Urinary catheters should be removed within 48 h of mid/lower rectal resections.	Removed after patient mobilization under the same conditions.

**Table 2:** Operative and Postoperative data.

Variables	Group Practices *n* = 256	Solo Practices *n* = 468	*p*
Operative procedure			0.474
Right hemicolectomy	62 (24.2%)	102 (21.8%)	
Left hemicolectomy	22 (8.6%)	35 (7.5%)	
Anterior resection	88 (34.4%)	159 (34.0%)	
Low anterior resection	54 (21.1%)	126 (26.9%)	
Others	30 (11.7%)	46 (9.8%)	
Combined surgery	18	24	0.320
Hepatectomy	4	12	0.441
Urology	5	5	0.336
Gynaecology	9	7	0.110
Blood loss < 50 mL	195 (76.2%)	357 (76.3%)	0.400
Surgical time (hh:mm)	4:09 ± 1:33	4:13 ± 1:30	0.539
Diagnosis			0.202
Malignancy	218 (85.2%)	414 (88.5%)	
Benign neoplasm	9 (3.5%)	15 (3.2%)	
Diverticular disease	7 (2.7%)	7 (1.5%)	
Constipation	7 (2.7%)	19 (4.0%)	
Others	15 (5.9%)	13 (2.8%)	
Cancer stage			0.282
Stage 0/I/II	109 (50%)	224 (54.1%)	
Stage III/IV	109 (50%)	190 (45.9%)	
NOSE	76 (29.7%)	107 (22.9%)	0.043
Operative method			<0.05
Laparoscopy	233 (91.0%)	466 (99.6%)	
Robotic-assisted	23 (9.0%)	2 (0.4%)	
Diverting stoma	24 (9.4%)	63 (13.5)	0.106
Morbidity	36 (14%)	100 (21.4%)	0.048
Grade II	28 (10.9%)	73 (15.6%)	
Grade III	8 (3.1%)	27 (5.8%)	0.113
Readmission	7 (2.7%)	16 (3.4%)	0.616
Discharge day			
≤4 days	73 (28.5%)	33 (7.1%)	<0.05
≤5 days	114 (44.5%)	104 (22.2%)	<0.05
≤6 days	162 (63.3%)	179 (38.2%)	<0.05
Discharge day (POD)			
Mean	6.6 ± 3.2	8.6 ± 5.5	<0.05
Median	6	7	
Postoperative blood test			
WBC ≥ 10^4^/dL	103 (40.2%)	158 (33.8%)	0.13
Hb ≥ 10 g/dL	195 (76.2%)	345 (73.7%)	0.489
CRP (mg/dL)	80.91 ± 51.15	73.88 ± 62.24	0.108
Pain score	2.25 ± 0.77	2.36 ± 0.68	0.048

NOSE: natural orifice specimen extraction; POD: postoperative day; WBC: white blood cell; Hb: hemoglobin; CRP: C reactive protein.

**Table 3:** Univariate and multivariate analysis of discharge within 5 days postoperatively.

Variables	Univariable Analysis		Multivariable Analysis	
	OR (95% CI)	*p*		*p*
Group practice	2.810 (2.022–3.905)	<0.001	2.836 (1.985–4.051)	<0.001
NOSE	3.790 (2.662–5.396)	<0.001	3.488 (2.333–5.096)	<0.001
Operative method		<0.001		0.24
Right hemicolectomy	REF		REF	
Left hemicolectomy	1.232 (0.651–2.331)	0.521	1.161 (0.578–2.331)	0.674
Anterior resection	1.450 (0.953–2.205)	0.083	1.170 (0.736–1.859)	0.507
Low anterior resection	0.610 (0.374–0.994)	0.047	0.608 (0.354–1.043)	0.071
Others	0.515 (0.264–1.005)	0.052	0.471 (0.224–0.991)	0.047
Robotic surgery	1.368 (0.580 × 3.226)	0.474		
Male	0.950 (0.690–1.307)	0.751	NS	
Age < 65 y	1.109 (0.804–1.529)	0.530	NS	
BMI > 25 kg/m^2^	1.313 (0.952–1.811)	0.096	NS	
Neoadjuvant therapy	0.445 (0.228–0.868)	0.018	0.721 (0.336–1.547)	0.401
ASA 3 (ref ASA 2)	0.809 (0.578–1.131)	0.215	NS	
Tumour > 4 cm	0.675 (0.481–0.949)	0.024	0.929 (0.614–1.404)	0.726
Stage		0.407	NS	
Benign disease	REF	0.818		
Early stage I/II	0.944 (0.579–1.539)	0.300		
Advanced stage III/IV	0.768 (0.466–1.265)			
Blood loss > 50 mL	0.388 (0.214–0.703)	0.002	0.504 (0.263–0.965)	0.039
Preop WBC >10 k	1.179 (0.696–1.999)	0.540	NS	
Preop Hb > 10	1.239 (0.762–2.017)	0.388	NS	
Preop Alb >3.5	2.071 (0.948–4.524)	0.068	1.873 (0.788–4.448)	0.155
Preop CEA > 5	0.503 (0.332–0.762)	0.001	0.654 (0.413–1.037)	0.071
Preop CRP > 5	0.593 (0.398–0.883)	0.010	0.834 (0.519–1.341)	0.454

NOSE: natural orifice specimen extraction; REF: reference; NS: not significant; BMI: body mass index; ASA: American Society of Anesthesiologists; WBC: white blood cell; Hb: hemoglobin; Alb: albumin; CEA: carcinoembryonic antigen; CRP: C reactive protein.

## Data Availability

Due to privacy and ethical concerns, details of the data and how to request access are available from the corresponding author.

## Supplementary Materials

The following supporting information can be downloaded at: https://www.mdpi.com/article/10.3390/jcm11236992/s1, Table S1: Comparison of the strategy between group practice and solo practice.
