# Supplementary material for: The Nuts and Bolts of Implementing a Modified ERAS Protocol for Minimally Invasive Colorectal Surgery: Group Practice vs. Solo Practice
Source: J Clin Med. 2022 Nov 26;11(23):6992. doi: 10.3390/jcm11236992 (PMC9739177; doi:10.3390/jcm11236992)
Supplement: Supplementary file 1 [file jcm-11-06992-s001.zip › jcm-2002770-supplementary.pdf]

Supplementary Table S1. Comparison of the strategy between group practice and solo practice

	Group practice	Solo practice
Members	4 attending surgeons<br>Senior and junior residents<br>Nurse practitioners	One attending surgeon<br>Senior and junior residents<br>Nurse practitioners
Leader	One of the four attending surgeons	The same one attending surgeon.
Ward round schedule	Regular and start at 8:30 every day.	Every day but irregular time.
Decision-making model	One of the four attending surgeons coordinates and makes decisions based on team input.<br>Communicate and record the consideration and objective.	A single attending surgeon makes the final decision on the patient's care.
Modified ERAS implementation	Rely on the modified ERAS protocol checklist.	According to single attending surgeon’s preference.
Clinical handover	Clear recording of diagnosis, complaints, assessments, goals, and plans is needed.<br>Structured records help organize documentation to guarantee nothing is missing.<br>An electronic note keeper is essential for clinical handoff and team communication.	Less necessary.
